# Lactylation in Vascular Diseases: A Double-Edged Sword

**DOI:** 10.3390/cells14241987

**Published:** 2025-12-14

**Authors:** Siyao Luo, Yafang Wang, Zhimo Luo, Aiguo Dai, Qing Dai

**Affiliations:** 1 School of Medicine, Hunan University of Chinese Medicine, Changsha 410208, China; luosiyao1284@stu.hnucm.edu.cn (S.L.); wangyafanglcyx@stu.hnucm.edu.cn (Y.W.); lwztmy1987623@stu.hnucm.edu.cn (Z.L.); daiaiguo@hnucm.edu.cn (A.D.); 2 Hunan Provincial Key Laboratory of Vascular Biology and Translational Medicine, Changsha 410208, China

**Keywords:** lactate, lactylation, vascular diseases, double-edged sword

## Abstract

**Highlights:**

**What are the main findings?**

- Lactate-derived lactylation functions as a key metabolic sensor that couples glycolytic flux with epigenetic remodeling and protein regulation in the vascular system. This review highlights the “double-edged sword” nature of lactylation across multiple vascular diseases.
- The pro-disease or protective effects of lactylation depend on disease stage and the cellular microenvironment.

**What is the implication of the main findings?**

- These findings deepen the understanding of lactylation as a critical epigenetic modification linking metabolic reprogramming with the pathophysiological mechanisms of vascular diseases.
- Targeting specific regulatory nodes within the lactate-lactylation process—including writers, erasers, and metabolic enzymes—provides a novel conceptual framework for developing diagnostic biomarkers and precision therapies for vascular diseases.

**Abstract:**

In recent years, lactate has transitioned from being considered a mere metabolic end-product to being regarded as a critical signaling molecule that links cellular metabolism with gene regulation. Protein lactylation, a post-translational modification (PTM) mediated by lactate, is central to this functional transformation. In vascular diseases, the lactate–lactylation process demonstrates a marked double-edged sword characteristic, with its regulatory effects highly dependent on cell type, disease stage, and the pathological microenvironment. On one hand, lactylation can exert protective roles by promoting reparative gene expression, driving anti-inflammatory cell polarization, and maintaining myocardial structural integrity; on the other hand, aberrant lactylation can exacerbate inflammatory responses, promote fibrosis, and induce cell death and vascular calcification, thereby driving the development and progression of atherosclerosis, heart failure, and stroke. This review systematically delineates the paradoxical yet unified dual roles of lactylation across various vascular diseases and explores the molecular bases that underlie these functional differences. We propose that deciphering and precisely modulating the ‘double-edged sword’ of lactylation—selectively enhancing its protective functions while suppressing its pathological actions—represents a central challenge and a critical opportunity for translating basic research into clinical applications. Such advances could provide a novel theoretical framework for the development of diagnostic biomarkers and cell-specific precision therapeutic strategies.

## 1. Introduction

Historically, lactate was simplistically defined as the terminal product of anaerobic glycolysis, and its accumulation under hypoxic or high-energy demand conditions was considered a hallmark of cellular stress. However, with the proposal of the lactate shuttle hypothesis, the scientific community began to re-evaluate the physiological roles of lactate, recognizing that it is not only an important energy substrate but also an important signaling molecule mediating metabolic communication between cells and tissues [1]. This conceptual shift culminated in 2019, when a landmark study using mass spectrometry first revealed lysine lactylation (Kla), demonstrating that glycolysis-derived lactate can directly serve as a substrate for a novel post-translational modification—histone lactylation—to regulate gene transcription [2].

Post-translational modifications (PTMs) are fundamental in expanding the functional diversity of proteins and regulating cellular signaling in human pathology. Well-established PTMs play pivotal roles in vascular homeostasis and disease: glycosylation significantly influences endothelial cell receptors and immune recognition [3]; methylation (both on DNA and histones) governs long-term epigenetic gene silencing or activation [4,5]; and carbonylation serves as a hallmark of irreversible oxidative damage commonly observed in atherosclerosis and vascular aging [6,7]. Unlike these classic PTMs, lysine lactylation represents a distinct “metabolic sensor” modification. It uniquely couples the cellular metabolic status (specifically glycolytic flux) directly to the epigenetic regulatory network, thereby offering a rapid and reversible response to metabolic stress. This core feature, distinct from traditional PTMs, not only fundamentally overturned the traditional understanding of lactate, but also endowed it with a new functional dimension as a molecular bridge linking cellular metabolic status and the epigenetic regulatory network, thereby filling an important gap in the metabolism–epigenetics intersection. Subsequent studies rapidly confirmed that lactylation is not limited to histones but is also widely present on non-histone proteins [8], profoundly affecting cellular activities by directly altering protein conformation, activity, stability, or interactions.

Vascular diseases broadly refer to pathological alterations affecting the structure and function of the circulatory system, which can lead to severe clinical manifestations and substantially reduce patients’ quality of life [9]. Cardiovascular diseases, as a representative group, are a leading cause of death worldwide. Moreover, other conditions closely associated with vascular pathology—such as cerebrovascular diseases, diabetic complications, and preeclampsia—also impose significant global burdens. In many vascular-related diseases, the pathophysiological processes are accompanied by marked metabolic reprogramming that results in enhanced glycolysis and substantial lactate accumulation. Therefore, lactylation, as a functional executor of this metabolic alteration, has garnered considerable attention. Preliminary studies indicate that lactylation plays a complex ‘double-edged sword’ role in vascular diseases: for example, in cardiovascular disease, it can both exacerbate pro-inflammatory responses and promote atherosclerosis [10], while also contributing to myocardial repair [11], indicating highly context-dependent mechanisms.

More importantly, the elucidation of this mechanism suggests that lactylation functions not merely as another post-translational modification but rather as a metabolic stress sensor. Intracellular lactylation levels directly reflect the intensity of glycolytic flux, thereby converting metabolic stresses (such as hypoxia and inflammation) in real time and quantitatively into distinct functional cellular responses. Under conditions of elevated glycolytic pressure, rising lactate concentrations drive the lactylation of key gene promoters or functional proteins, executing corresponding transcriptional or proteomic programs. This direct metabolism–epigenetics coupling renders lactylation a unique entry point for understanding vascular disease pathophysiology.

Although recent reviews have provided valuable overviews of lactylation in cardiovascular diseases, cancer, general inflammation, or broad metabolic disorders, there remains a lack of a dedicated, systematic review focusing specifically on lactylation within the vascular system. To address this gap, we integrated cardiovascular, cerebrovascular, and peripheral vascular diseases—including preeclampsia and diabetic vasculopathy—under a unified mechanistic framework of “lactate–lactylation.” We then systematically categorized the context-dependent and cell-type-specific dual roles of lactylation across disease stages, distinguishing its protective reparative functions from its detrimental effects. Moreover, we comprehensively summarized the latest evidence on the emerging frontier of non-histone lactylation, such as modifications of metabolic enzymes and cytoskeletal proteins, thereby providing a new molecular basis for understanding the broad regulatory scope of lactylation.

By focusing on the vascular system, this review summarizes recent advances regarding the dual regulatory roles of the lactate–lactylation process across various vascular diseases, aims to clarify the molecular biological basis of lactylation, and explores how it functions as a universal regulatory module governing immune inflammation, tissue remodeling, cell fate decisions, and other core pathological processes, thereby providing new perspectives for research in this field.

## 2. Search Strategy and Selection Criteria

To provide a comprehensive overview of the role of lactylation in vascular diseases, we conducted a literature search in major databases, including PubMed and Web of Science, up to September 2025. The search keywords included combinations of “lactate”, “lactylation”, “histone lactylation”, “vascular diseases”, “atherosclerosis”, “myocardial ischemia–reperfusion injury”, “heart failure”, “stroke”, “hypertension”, and “diabetic vascular complications”. We primarily focused on peer-reviewed original research articles and reviews published after 2019, following the initial identification of histone lactylation, although seminal papers on lactate metabolism were also included. This article is structured as a narrative review to synthesize current findings, mechanisms, and translational prospects of the lactate–lactylation process in vascular pathologies.

## 3. Overview of Lactylation

### 3.1. Discovery of Lactylation

In 2019, Zhang et al. used mass spectrometry to confirm the existence of lysine lactylation, first revealing histone lactylation as a novel epigenetic modification. Their team demonstrated that modulating the activity of lactate dehydrogenase during glycolysis affects lactate production and thereby regulates histone lactylation, indicating that histone lactylation is a new enzymatic post-translational modification [2]. Beyond histone lactylation, subsequent studies have shown that lactylation also widely occurs in non-histone proteins. Gaffney and colleagues found that lactylation targets multiple glycolytic enzymes to form a negative feedback loop that regulates glycolysis, thereby affecting cellular metabolism. Moreover, their work suggested that lactylation can occur via non-enzymatic acyl transfer mediated by lactoyl-glutathione (LGSH) [8]. Since the identification of lactylation in 2019, its roles in various vascular diseases have rapidly become a research focus. Notably, in 2022, Wang and colleagues first demonstrated that histone lactylation facilitates myocardial repair after myocardial infarction [11], opening the field to subsequent discoveries of lactylation’s critical roles in atherosclerosis, heart failure, stroke, and other vascular conditions.

### 3.2. Mode of Lactylation

Lactylation can be categorized into L-lactylation and D-lactylation based on the stereoisomers of lactate. L-lactate is the primary product of glycolysis, whereas D-lactate is generally a minor byproduct of metabolic pathways [12]. L-lactylation uses L-lactate as the precursor, with lactyl-CoA serving as the acyl donor in an enzymatic lysine lactylation process [2]. Currently, L-lactylation is the most extensively studied type of lactylation and occurs prominently under conditions such as hyperglycemia and hypoxia [13,14]. It has been demonstrated to play essential roles in cardiovascular pathophysiology, including inhibiting cardiomyocyte apoptosis, alleviating excessive inflammation, and promoting vascular regeneration [15,16]. In contrast, D-lactylation originates from methylglyoxal (MGO). During glycolysis, a portion of glyceraldehyde-3-phosphate and dihydroxyacetone phosphate (DHAP) can be non-enzymatically converted into the glycolytic byproduct MGO. MGO is then converted into lactoylglutathione (LGSH) by glyoxalase 1 (GLO1) with the assistance of glutathione [17,18]. LGSH derived from MGO is subsequently hydrolyzed by GLO2 to produce D-lactate and glutathione [18]. The lactyl group from MGO-derived LGSH is transferred to lysine residues, resulting in D-lactylation, also referred to as non-enzymatic lysine lactylation [8]. Studies have shown that D-lactate itself exerts immunomodulatory and neuroprotective functions in diseases such as cancer and cerebral ischemia [19,20], suggesting that D-lactylation may also possess important and yet underexplored biological functions. It is currently believed that both L- and D-lactylation pathways can coexist within the same cell. Additionally, L-lactate may inhibit LGSH degradation, increasing its level and thus promoting D-lactylation, suggesting potential crosstalk between the two modifications. Although the role of L-lactylation in cardiovascular diseases has begun to be elucidated, the specific functions of D-lactylation—and whether interactions between these two modifications occur in vascular and other diseases—remain largely unknown.

### 3.3. Regulatory Mechanisms of Lactylation

Lactylation levels increase in a dose-dependent manner with lactate concentration and are primarily regulated through three core processes: lactate production and transport, lactyl transferases, and enzymes responsible for lactate clearance.

#### 3.3.1. Lactate Production Promotes Lactylation

Lactylation levels increase in a dose-dependent manner with lactate concentration. Histone lactylation was initially considered to be triggered by endogenous lactate. Subsequent studies have shown that both exogenous sodium lactate and elevated endogenous lactate levels can promote histone lactylation, with endogenous lactate having a more pronounced effect [2,21]. Thus, lactate generation and transport are key determinants of lactylation. As lactate is the terminal product of glycolysis, its generation depends on the balance between glycolysis and mitochondrial oxidative phosphorylation. Thus, alterations in the activity of key glycolytic enzymes can significantly affect lactate production and thereby modulate lactylation levels. For example, during M1 macrophage polarization, knockdown of the lactate dehydrogenase A (LDHA) α-subunit reduces macrophage lactate concentrations and global histone lactylation levels [2]. Lactate production depends on the balance between glycolysis and the TCA cycle, with enhanced glycolytic activity typically leading to increased lactylation levels. Inhibition of mitochondrial respiration by rotenone suppresses the tricarboxylic acid cycle, increases lactate accumulation, and elevates histone lactylation [22,23]. In contrast, the non-metabolizable glucose analog 2-deoxy-D-glucose (2-DG) inhibits glycolysis and markedly reduces lactylation levels [24,25].

#### 3.3.2. Lactate Transport Affects Lactylation

Lactate transport is another key factor influencing lactate levels and lactylation. As mentioned above, lactate exists as two stereoisomers—L-lactate and D-lactate—both of which are shuttled across cellular compartments by the monocarboxylate transporter (MCT) family [26]. MCTs play key roles in extracellular lactate uptake and intracellular lactylation processes. L-lactate can be converted to acetyl-CoA and participate in lysine lactylation via acetyl transfer mechanisms. The transport of L-lactate is mainly mediated by MCT1 and MCT2, which facilitate its influx from the extracellular space, while MCT4 promotes its efflux, regulated by the lactate concentration gradient [27,28,29]. In the tumor microenvironment, hypoxic cancer cells produce lactate through LDHA and release it into the extracellular space via MCT4. Subsequently, normoxic cells take up lactate via MCT1 and convert it to pyruvate through LDHB, generating ATP [30]. In sepsis, macrophages import extracellular lactate through MCTs, which mediates lactylation of HMGB1. Monocarboxylate transporter inhibitors can suppress MCT activity, reducing macrophage uptake of extracellular lactate and decreasing HMGB1 lactylation levels [31]. For cells with low endogenous lactate production, MCTs may play a more critical role in driving lactylation.

#### 3.3.3. Lactyltransferases and Lactate Removing Enzyme of Lactylation

In enzymatic pathways, lactate is believed to be converted into lactoyl-CoA via lactoylation catalyzed by CoA-transferase [2]. Enzymes responsible for transferring the lactyl group are likely key regulators of the extent of lactylation. Multiple enzymes involved in converting lactate to lactoyl-CoA have been identified in microbes, such as YdiF in Escherichia coli and ME-PCT in Megasphaera elsdenii [17,32]. Recent studies show that alanyl-tRNA synthetases AARS1 and AARS2 are bona fide lactoyltransferases, functioning as L-lactate sensors by binding intracellular L-lactate and directly transferring the lactoyl group to lysine residues, thereby inducing lysine lactoylation [33,34,35]. Notably, acetyl-CoA synthetase 2 (ACSS2) has been identified as a lactyl-transferring enzyme that mediates histone lactylation and promotes tumor immune escape [36]. Furthermore, E1A binding protein p300 (EP300) and CREB-binding protein (CBP) have been reported to act as lactyl transferases catalyzing the transfer of lactyl moieties to lysine residues [2,31]. Knockout of EP300 in human colorectal cancer HCT116 and HEK293T cells reduces global histone lactylation and H3K18la levels [2]. Conversely, Varner et al. reported that histone deacetylases HDACs can serve as erasers for lysine lactylation by catalyzing removal of ε-N-lactyl modifications. Moreno-Yruela and colleagues further demonstrated that HDAC1–3 efficiently remove lysine lactylation, while class III HDACs (SIRT1–3) also possess lactylation-deacylase activity [37,38].

### 3.4. Pathophysiological Roles of Lactylation

#### 3.4.1. Lactylation and Tissue Repair

Lactylation acts as a molecular switch in tissue repair, governing post-injury reparative processes by regulating gene expression and cellular functions, thereby leading to recovery. During the inflammatory reparative phase, Irizarry-Caro et al. demonstrated that lactate-mediated histone lactylation promotes reparative gene expression and facilitates the transition of macrophages from an inflammatory phenotype to a reparative phenotype, which is critical for tissue repair [39]. Wang et al. found that lactate promotes lactylation at lysine 62 of pyruvate kinase M2 (PKM2 K62la), enhancing PKM2 activity and driving the shift of pro-inflammatory macrophages toward a reparative phenotype, thereby accelerating wound healing [40]. This mechanism appears to be conserved across different tissue repair scenarios. In skin wound healing, Wei et al. reported that myeloid-expressed TREM2 activates the Akt/mTOR/HIF-1α pathway to promote macrophage glycolysis and lactate production, which in turn stabilizes HIF-1α through lactylation, promoting VEGF and other pro-angiogenic factors to enhance angiogenesis and wound repair [41]. In spinal cord injury (SCI), Hu et al. also observed the potential mechanism of lactylation in which lactate-mediated H4K12la promotes PD-1 transcription in microglia; the lactate/H4K12la/PD-1 axis then promotes microglial proliferation, axonal regeneration, and motor recovery, aiding SCI repair [42]. Wu et al. also reported that lactylation can restore osteogenic differentiation capacity of periodontal ligament stem cells in periodontitis, supporting periodontal tissue repair [43].

#### 3.4.2. Lactylation and Cellular Metabolism

Extensive studies have revealed close associations between lactylation and multiple aspects of cellular metabolism. As the end-product of glycolysis, lactate can mediate lactylation to regulate gene expression and protein function, thus influencing glucose and lipid metabolism, with differential effects in physiological regulation and disease progression.

In glucose metabolism, lactylation can modulate glycolysis and the pentose phosphate pathway (PPP) through various mechanisms. Pan et al. found that in microglia isolated from the brains of 5×FAD mice, both lactate and H4K12la levels were significantly increased; H4K12la was enriched at promoters of HIF-1α, PKM, and LDHA, activating their expression and thereby enhancing glycolytic activity and consequently aggravating microglial dysfunction in Alzheimer’s disease (AD) [44]. In colorectal cancer, Chen et al. reported that l metabolic reprogramming leads to excessive lactate accumulation, which not only promotes NSUN2 expression through H3K18 lactylation, but also enhances the RNA-modifying capacity of NSUN2 via lactylation at lysine 356. This modification upregulates ENO1 expression, accelerates glycolytic flux, and ultimately results in poor clinical outcomes [45]. Notably, lactate generated from the Warburg effect drives protein lactylation; conversely, lactylation further sustains high glycolytic activity by modifying key enzymes (such as PKM2) or transcription factors. This bidirectional “metabolism–epigenetics” regulatory loop forms a positive feedback cycle that continuously reinforces metabolic rewiring and functional transformation, thereby accelerating disease progression [46]. In the PPP, Meng et al. observed in cervical cancer that lactylation at DCBLD1 K172 promotes DCBLD1 expression and inhibits autophagic degradation of glucose-6-phosphate dehydrogenase (G6PD), resulting in activation of the PPP [47]. Conversely, in HPV16 E6-driven cervical cancer, reduction in lactate production suppressed G6PD K45 lactylation, increasing G6PD enzymatic activity and similarly activating the PPP, which contributes to cancer progression [48].

Regarding lipid metabolism, lactylation plays a pivotal role by either inhibiting or promoting lipid synthesis. Chen et al. showed that high-intensity interval training increases lactate levels that induce lactylation of fatty acid synthase (FASN), which in turn suppresses FASN activity and consequently reduces fatty acid synthesis [49]. In non-alcoholic fatty liver disease (NAFLD), Gao et al. found that inhibition of mitochondrial pyruvate carrier 1 (MPC1) elevates lactate levels and promotes FASN K673 lactylation, which similarly suppresses FASN activity and reduces lipid synthesis, ameliorating hepatic lipid accumulation in NAFLD [50]. Additionally, Yin et al. reported that the traditional Chinese medicinal formulation Huazhuo Tiaozhi granules ameliorates dyslipidemia by regulating lactylation of histone H2BK6 and H4K80, leading to downregulation of miR-155-5p and suppression of lipid synthesis [51]. Wang’s group further demonstrated that under nitrogen stress, lactylation of acetyl-CoA carboxylase promotes lipid synthesis in microalgae; lactylation also regulates transcription and protein activities of lipid metabolism-related factors ACP, KAS, and ACDH, and it activates the TCA cycle and β-oxidation to facilitate lipid biosynthesis [52,53].

#### 3.4.3. Lactylation and Immune Regulation

Lactylation displays bidirectional effects in immune regulation: in many contexts, it promotes inflammation and immune evasion to advance disease progression, whereas in specific situations it can suppress excessive immune activation and maintain immune homeostasis.

In autoimmune diseases, Fu et al. used single-cell sequencing to show that plasma cells from rheumatoid arthritis (RA) patients exhibit elevated RA lactylation scores (RAlac_score), which positively correlate with immune cell infiltration and expression of immune checkpoint molecule, suggesting lactylation as a potential diagnostic and prognostic marker for RA [54]. In experimental autoimmune uveitis (EAU), Fan et al. found that lactylation at Ikzf1 K164 in CD4^+^ T cells directly regulates expression of Th17-promoting genes Runx1, Tlr4, facilitating Th17 differentiation and exacerbating EAU inflammation and progression [55]. Zhang et al. reported that in systemic lupus erythematosus (SLE) patients’ monocytes, lactate promotes lactylation of cGAS, inhibiting its interaction with the E3 ubiquitin ligase MARCHF5, blocking cGAS degradation and resulting in persistent type I interferon (IFN-1) production that drives disease progression [56].

Lactylation also plays an important role in immunosuppression and immune escape. Tong et al. observed in colorectal cancer that tumor cells use lactylation to stabilize programmed death-ligand 1 (PD-L1) expression, reducing CD8^+^ T cell efficacy and promoting immune evasion [57]. Wang et al. found in head and neck squamous cell carcinoma that lactate accumulation induces H3K9la to promote IL-11 expression and activate the JAK2/STAT3 pathway, upregulating CTLA-4 and PD-1 while downregulating IFN-γ and GZMB, thereby impairing CD8^+^ T cell function and facilitating immune escape [58]. Gu et al. identified lactylation of MOESIN at K72, induced by lactate in the tumor microenvironment (TME), as a mechanism that enhances its binding to TGF-β receptor I. This binding activates TGF-β signaling, which in turn upregulates FOXP3 expression and reinforces Treg-mediated immunosuppression [59]. Xiong et al. further demonstrated that lactate in TME induces H3K18la to upregulate METTL3, whose K281 and K345 sites are lactylated; this enhances METTL3-mediated m6A modification of Jak1 mRNA, activating JAK–STAT3 signaling and promoting expression of immunosuppressive genes such as IL-6, thereby augmenting immunosuppressive functions of tumor-infiltrating myeloid cells [60].

Conversely, lactylation can suppress inflammation and contribute to immune homeostasis in certain contexts. Lactate-treated Th17 cells exhibit increased H3K18 lactylation, which suppresses the pro-inflammatory functions of Th17 cells and promotes their conversion toward anti-inflammatory Treg-like phenotypes [61]. Li et al. showed that lactylation mediated by AARS2 inactivates cGAS, thereby inhibiting innate immune responses [33]. The metabolite S-D-lactoylglutathione (SLG) can directly induce cytoplasmic protein lactylation in immune cells, notably at RelA K310, which suppresses inflammatory signaling and NF-κB transcriptional activity, consequently limiting immune activation and restoring homeostasis [62].

#### 3.4.4. Lactylation and Autophagy

Sun et al. identified multiple core autophagy proteins modified by lactylation—such as PIK3C3/VPS34, ULK1, and UVRAG—by mass spectrometry, immunoprecipitation, and Western blotting, indicating an important role for lactylation in autophagy regulation [63]. Accumulating evidence indicates that lactylation can exert dual effects on disease progression by modulating autophagy.

Jia et al. revealed that lactate-mediated lactylation of Vps34 increases its lipid kinase activity, promoting autophagosome formation and maturation; in skeletal muscle cells, Vps34 lactylation enhances autophagy, which supports muscle homeostasis [64]. Xu et al. observed that histone lactylation, particularly H3K18la, is upregulated following traumatic brain injury (TBI); ChIP–qPCR showed enrichment at the PSMD14 promoter, upregulating PSMD14 expression, promoting mitophagy, reducing ROS production, and suppressing neuronal PANoptosis, thereby improving outcomes after TBI [65].

In contrast, Li et al. reported that H3K18la can promote transcription of the autophagy enhancer RUBCNL, activating autophagy and facilitating survival and proliferation of colorectal cancer cells in hypoxia [25]. Deng et al. found that in bladder cancer H3K18la enhances PRKN expression, promoting mitophagy and M2 macrophage polarization to support tumor progression [66]. Zhang et al. showed that lactate upregulates AMPKα lactylation while downregulating AMPKα phosphorylation, inhibiting AMPK signaling and autophagy and promoting cellular senescence, thereby accelerating intervertebral disk degeneration [67]. Wang et al. observed in a cigarette smoke (CS)-induced AD model that CS-induced H4K12la activates transcription of the NLRP3 inflammasome and suppresses microglial autophagy via mTOR signaling, contributing to AD pathology and cognitive decline [68]. An et al. reported that Tau protein lactylation modulates ferritinophagy in AD: Tau K677 lactylation, through the MAPK pathway, increases expression of ferritinophagy-related proteins NCOA4, ATG5, Beclin1, and LC3II, promoting ferritinophagy and ferroptosis, as well as exacerbating AD progression [69]. Li et al. found that lactylation of aldehyde dehydrogenase 2 (ALDH2) disrupts its interaction with the mitophagy receptor prohibitin 2 (PHB2), promoting PHB2 ubiquitination and proteasomal degradation, thereby inhibiting mitophagy and worsening mitochondrial dysfunction in acute kidney injury [70]. Weng et al. detected increased non-histone lactylation after TBI, notably at mitochondrial translation elongation factor Tufm K286, which inhibits Tufm transport from the cytosol to mitochondria, reducing mitochondrial Tufm and impairing Tufm-mediated mitophagy, thereby promoting neuronal apoptosis and aggravating TBI [71].

#### 3.4.5. Lactylation and Fibrosis

**Figure 1:**
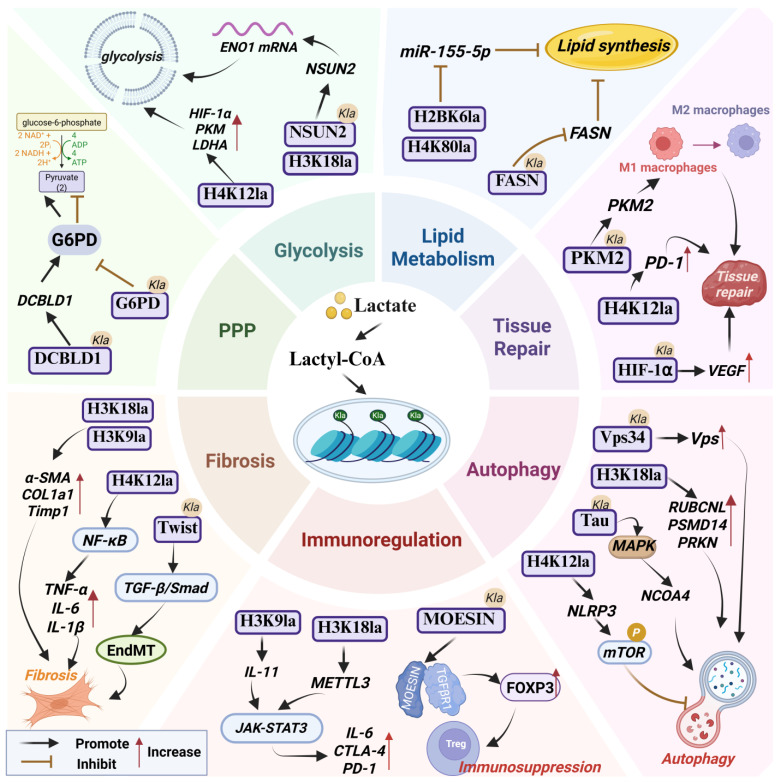
The pathophysiological role of lactylation. Abbreviations used in the figure: Ctla4, Cytotoxic T-Lymphocyte-Associated Protein 4; DCBLD1, Discoidin, CUB and LCCL Domain-Containing Protein 1; EndMT, Endothelial–Mesenchymal Transition; FASN, Fatty Acid Synthase; G6PD, Glucose-6-Phosphate Dehydrogenase; NCOA4, Nuclear Receptor Coactivator 4; PKM2, Pyruvate Kinase M2; PPP, Pentose Phosphate Pathway; Vps34, Vacuolar Protein Sorting 34.

Lactylation drives fibrosis across various organs through multiple mechanisms. In liver fibrosis, Rho et al. demonstrated that hexokinase 2 (HK2) in activated hepatic stellate cells (HSCs) induces lactate production; histone lactylation, particularly H3K18la, then promotes expression of α-SMA, COL1A1, and TIMP1, advancing liver fibrosis. They also observed a competitive relationship between H3K18 acetylation and lactylation, where dynamic balance between these modifications influences HSC activation and fibrogenesis [72]. Zhou et al. reported that insulin-like growth factor 2 mRNA-binding protein 2 (IGF2BP2), a novel m6A reader, promotes H3K18la via glycolytic regulation to activate HSCs and accelerate liver fibrosis [73]. Similarly, Wu et al. showed that LDHA-mediated H3K18la promotes SOX9 transcription and accelerates liver fibrosis [74]. In renal fibrosis, Wang et al. found that PFKFB3-driven lactate accumulation promotes H4K12la, which activates NF-κB signaling and upregulates fibrotic factors such as α-SMA and CTGF, thereby driving renal fibrosis [75]. Xiang et al. reported that overexpression of PKM2 leads to lactate accumulation and histone lactylation, activating the TGF-β1/Smad3 pathway and promoting renal fibrosis in chronic kidney disease [76]. Xu et al. observed that in ischemic flap fibrosis, aberrant PKM2 expression leads to excessive lactate production, promoting Twist1 lactylation and activating TGF-β/Smad2 signaling to drive fibrotic progression [77]. In intestinal fibrosis, Liu et al. found that downregulation of glucagon receptor (GCGR) and glucagon-like peptide-1 receptor (GLP1R) causes lactate accumulation, promotes H3K9la, and upregulates expression of fibrotic genes such as TGFβ1, thereby exacerbating intestinal fibrosis [78] (Figure 1).

## 4. Lactylation in Vascular Diseases: A Double-Edged Sword

**Figure 2:**
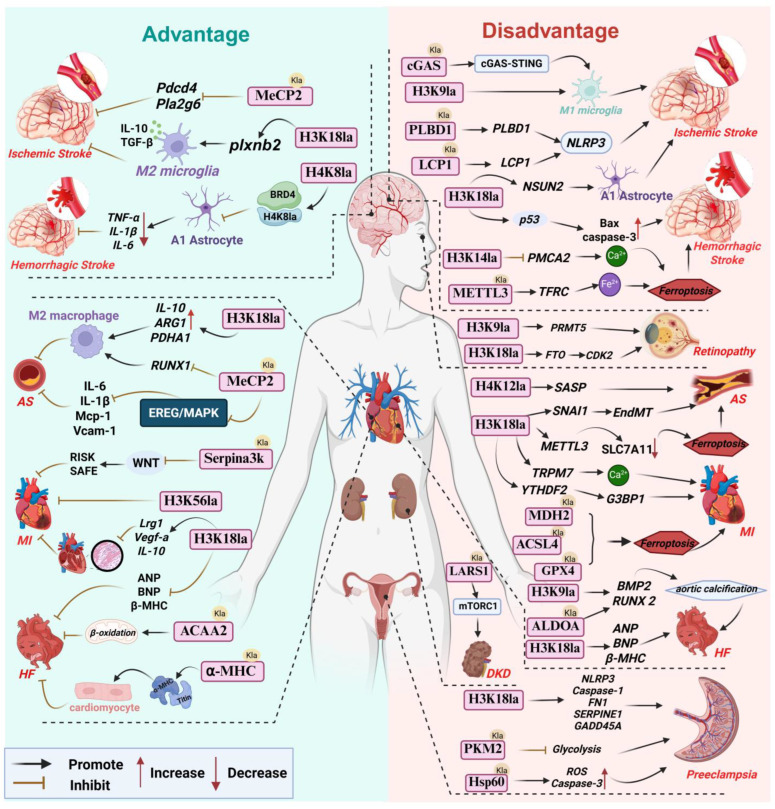
Lactylation: A double-edged sword in vascular diseases. Abbreviations used in the figure: AS, Atherosclerosis; α-MHC, α-Myosin Heavy Chain; ALDOA, Aldolase A; ACSL4, Acyl-CoA Synthetase 4; ACAA2, Acetyl-CoA Acyltransferase 2; BRD4, Bromodomain Containing 4; BMP2, Bone Morphogenetic Protein 2; cGAS, Cyclic GMP-AMP Synthase; DKD, Diabetic Kidney Disease; EndMT, Endothelial-Mesenchymal Transition; FN1, Fibronectin 1; GPX4, Glutathione Peroxidase 4; HF, Heart Failure; LARS1, Leucyl-tRNA Synthetase 1; LCP1,Lymphocyte Cytosolic Protein 1; MDH2,Malate Dehydrogenase 2; MeCP2, Methyl-CpG Binding Protein 2; MI, Myocardial Infarction; Pdcd4, Programmed Cell Death 4; plxnb2,Plexin B2; SASP, Senescence-Associated Secretory Phenotype; SLC7A11,Solute Carrier Family 7 Member 11; TFRC, Transferrin Receptor; YTHDF2, YTH N6-Methyladenosine RNA Binding Protein 2.

For a long time, lactate was widely viewed as the metabolic endpoint of glycolysis. However, recent studies indicate that lactate not only serves as an energy substrate that can be reutilized but also functions as an important signaling molecule. Lactylation, as a mechanism mediating its biological functions, has consequently garnered increasing attention. Notably, lactylation can be classified into L-lactylation and D-lactylation depending on lactate stereoisomers. Current research in the cardiovascular field predominantly focuses on ischemia-induced L-lactylation (enzymatic lactylation), whereas the potential significance of D-lactylation (non-enzymatic lactylation) in metabolic vascular diseases should not be overlooked. In the progression of vascular diseases, lactylation behaves as a double-edged sword with bidirectional regulatory roles. Under physiological or mild stress conditions, it can exert protective effects by modulating inflammatory responses and maintaining metabolic homeostasis; conversely, in persistent pathological states, excessive or aberrant lactylation can amplify inflammatory cascades, promote fibrosis and vascular remodeling, and thereby drive disease progression (Figure 2).

### 4.1. Cardiovascular Disease

#### 4.1.1. Atherosclerosis

Atherosclerosis is a chronic inflammatory disease driven by dysregulated lipid metabolism, characterized by lipid deposition in the arterial intima, foam cell formation, and inflammatory cell infiltration, ultimately leading to plaque formation and vascular lesions that pose serious threats to human health [79]. Studies have shown that plaque formation is closely associated with metabolic abnormalities in vascular smooth muscle cells (VSMCs), macrophages, and endothelial cells (ECs). Notably, macrophage polarization and lipid metabolic dysregulation in atherosclerosis exert reciprocal influences on each other. On the one hand, aberrant lipid metabolism modulates macrophage polarization and their anti-/pro-inflammatory properties; on the other hand, polarization status further affects lipid metabolic pathways [80]. For example, during M2 macrophage polarization, the expression of miR-125a-5p is downregulated, releasing its inhibitory effect on soluble VEGF receptor-1 (sVEGFR1) and thereby increasing its expression [81]. This suppresses VEGF-mediated transcription of genes involved in lipid uptake and storage, ultimately influencing lipid metabolism and potentially contributing to atherosclerosis progression. Moreover, hypoxic and glycolytic microenvironments in atherosclerotic lesions promote lactate accumulation, and lactylation may serve as a key regulatory mechanism linking macrophage polarization, metabolic reprogramming, and the pathophysiology of atherosclerosis.

Beneficial roles of lactylation in anti-atherosclerosis. Zhang and colleagues demonstrated that inhibition of MCT4 increases intracellular lactate levels in macrophages, which upregulates p300-mediated H3K18 lactylation. H3K18la becomes enriched at promoters of reparative genes such as IL-10 and PDHA1, promoting macrophage polarization from a pro-inflammatory M1 phenotype toward a reparative M2 phenotype, significantly reducing plaque area in HFD-fed ApoE^−/−^ mice and slowing disease progression. Clinical sample analysis also revealed a pronounced increase in MCT4 in atherosclerotic plaques, and experiments using human peripheral blood mononuclear cells (PBMCs) further validated that H3K18la may attenuate inflammation and reverse atherosclerosis through the above mechanisms [82]. Physical exercise has long been recognized as an effective strategy for preventing and treating atherosclerosis, and part of its mechanism may be mediated by lactylation. Wang et al. [16] reported that exercise increases lactate production and promotes lactylation of methyl CpG binding protein 2 (MeCP2) at K271. ChIP-qPCR results indicated that MeCP2 K271la accumulates at the Ereg promoter region, repressing Ereg expression and blocking the EREG/MAPK signaling pathway, thereby downregulating endothelial inflammatory mediators IL-6, IL-1β, VCAM-1 and MCP-1 as well as slowing plaque progression. In aortic plaques of exercise-intervened ApoE^−/−^ mice, enhanced MeCP2 K271la in macrophages promoted interaction with H3K36me3, induced demethylation of H3K36me3, reduced RUNX1 expression, upregulated M2 markers ARG1 and IL-10, downregulated M1 markers iNOS and TNF-α, and promoted macrophage polarization toward an M2 phenotype, increasing plaque stability [83] (Table 1).

Detrimental roles of lactylation in promoting atherosclerosis. Dong et al. [84] reported that oxidized LDL (ox-LDL) can induce aberrant aerobic glycolysis in ECs, causing lactate accumulation and elevated H3K18la levels. In a p300/ASF1A-assisted manner, H3K18la is enriched at the SNAI1 promoter, promoting SNAI1 expression and driving endothelial-to-mesenchymal transition (EndMT). Compared with non-atherosclerotic controls, atherosclerotic patient tissues showed higher H3K18la, decreased VE-cadherin and eNOS expression, as well as increased fibronectin-1 and collagen-1A expression, accelerating atherosclerosis progression. Chen et al. further revealed that ox-LDL stimulation upregulates HK2 and PKM2 activities in macrophages, inducing lactate accumulation and enhancing H3K18la, which in turn promotes METTL3 expression and—via a YTHDF2-dependent m6A mechanism—accelerates degradation of SLC7A11 mRNA, triggering ferroptosis and increasing release of inflammatory cytokines IL-1β, IL-6 and TNF-α, ultimately contributing to plaque formation [10]. VSMC senescence impairs plaque repair and compromises plaque stability. Li and colleagues found that in Ras-induced senescent VSMCs, TRAP1 promotes expression of phosphofructokinase-1 (PFK1), enhancing glycolysis, causing lactate accumulation and suppressing HDAC3 expression, thereby increasing H4K12la. ChIP-qPCR confirmed H4K12la enrichment at promoters of senescence-associated secretory phenotype (SASP) genes, driving SASP transcription in VSMCs and exacerbating VSMC senescence and atherosclerosis progression. In an ApoE^−/−^Trap1SMCKO mouse model, immunofluorescence showed reduced expression of senescence marker p21 and H4K12la, decreased plaque area and increased plaque stability; clinical samples also revealed elevated H4K12la and p53/p21/p16 protein levels in aortic tissues of atherosclerotic patients, suggesting involvement in disease progression [85].

Taken together, the role of lactylation in atherosclerosis underscores its cell-type and microenvironment-dependent complexity. Particularly, histone H3K18la functions a critical epigenetic mark and a central regulatory node: in macrophages, H3K18la induced by exercise or MCT4 inhibition tends to drive an anti-inflammatory, reparative M2 phenotype and thus exerts protective effects that suppresses plaque progression; however, in VSMCs and ECs, H3K18la triggered by pathological stimuli such as ox-LDL primarily promotes osteogenic differentiation, EndMT, and ferroptosis, accelerating atherogenesis. This duality likely stems from differences in chromatin accessibility between cell types. In macrophages, H3K18la is preferentially enriched at genomic regions encoding anti-inflammatory and metabolic repair genes. In contrast, within ECs, H3K18la is predominantly bound to gene loci associated with EndMT, such as SNAI1, in the absence of accessible anti-inflammatory genes, thereby contributing to detrimental effects. This underscores the critical necessity for future therapeutic interventions to be highly cell-specific. Current research is concentrated on histone modifications, whereas critical non-histone lactylation networks (e.g., MeCP2 lactylation) and their cell-specific functions remain to be elucidated—an important direction for precisely targeting lactylation in atherosclerosis.

#### 4.1.2. Myocardial Ischemia–Reperfusion Injury (MIRI)

Myocardial infarction (MI) is an acute life-threatening syndrome characterized by severe and sustained ischemia–hypoxia of cardiomyocytes leading to necrosis. Reperfusion therapy is the key lifesaving intervention for acute MI patients [86]. However, restoration of blood flow can paradoxically exacerbate myocardial injury [87], resulting in myocardial ischemia–reperfusion injury (MIRI).

In the early stage of MI, Wang et al. demonstrated in vitro that lactate raises H3K18la levels and enriches this mark at promoters of reparative genes such as Lrg1, Vegf-a and IL-10 in macrophages, promoting their transcription and thereby enhancing both anti-inflammatory and pro-angiogenic repair activities that facilitate cardiac repair post-MI. Their in vivo mouse experiments also showed that enhancing histone lactylation suppresses inflammatory cell infiltration, promotes angiogenesis and improves cardiac function [11]. Yu et al. [88] identified H3K56la as a key marker in rat cardiomyocytes under hypoxia/reoxygenation (H/R) stress: mechanistically, HSPA12A upregulates Smurf1 expression to maintain HIF-1α stability, activating glycolytic genes LDHA and HK2, promoting lactate accumulation and inducing H3K56la, thereby alleviating MI/R injury. Wang and colleagues [15] further noted that accumulated lactate in MIRI can lactylate SERPINA3K at K351 in cardiac fibroblasts, inhibiting WNT signaling and activating the RISK and SAFE pathways, which suppress cardiomyocyte apoptosis and reduce infarct size.

Lactylation also contributes to MIRI exacerbation. Fang et al. [89] demonstrated that in H/R-treated cardiomyocytes and mouse I/R models, upregulated LDHA increases lactate production and induces lactylation of NLRP3 at K245, which stabilizes NLRP3 and promotes inflammasome activation, increasing IL-1β and IL-18 release as well as expression of pyroptosis markers cleaved caspase-1, GSDMD-N, thereby exacerbating cardiomyocyte pyroptosis. Hu et al. [90] reported that H/R-induced glycolysis enhances histone lactylation, particularly H3K18la; ChIP-qPCR indicated H3K18la enrichment at the TRPM7 promoter, promoting TRPM7 transcription and expression, causing intracellular Ca^2+^ overload, Bax/Bcl-2 imbalance, and cardiomyocyte apoptosis; I/R mouse myocardium also exhibited increased serum markers LDH, CK-MB, and cTnI, indicating aggravated myocardial injury. Xu et al. [91] found that lactate promotes YTHDF2 transcription via EP300-mediated H3K18la, upregulating G3BP1 expression and inducing pathological cardiomyocyte hypertrophy and apoptosis, thereby worsening MIRI. However, inhibition of the “writer” EP300 significantly reduces lactylation, decreases YTHDF2 expression, and alleviates cardiomyocyte injury, indicating that lactylation directly regulates the role of YTHDF2 in MIRI. Following myocardial injury, sustained inflammation and metabolic remodeling can activate fibroblasts and promote their differentiation into myofibroblasts, leading to cardiac fibrosis after myocardial infarction. This is a multifactorial process involving complex signaling mediated by microRNAs, cytokines, and chemokines [92]. Fan et al. [93] showed that lactate induces lactylation of Snail1, enhancing its binding to TGF-β genes, upregulating TGF-β expression and activating the TGF-β/Smad2 pathway, promoting EndoMT and aggravating post-MI cardiac fibrosis and dysfunction. Although abundant evidence has established the importance of lactylation in MIRI, direct mechanistic studies focusing on lactylation in cardiac fibrosis remain limited and require further exploration.

Lactylation can also aggravate MIRI by modulating ferroptosis. She et al. [94] reported that in MIRI, enhanced glycolysis and suppressed oxidative phosphorylation cause lactate accumulation that induces lactylation of malate dehydrogenase 2 (MDH2) at K241. Using in vivo rat models and cardiomyocytes harboring point mutations at MDH2 K241 to mimic lactylated and delactylated states, the authors showed that MDH2 K241la disrupts mitochondrial structure and function, enlarges infarct size, and downregulates GSH and GPX4, thereby promoting ferroptosis and aggravating MIRI. In contrast, delactylation at MDH2 K241 improves cardiac function and reduces infarct size in I/R rats. Lv et al. [95] showed that lactylation of ACSL4 at K83 inhibits its ubiquitin-dependent degradation, increasing protein stability and exacerbating lipid peroxidation and ferroptosis; increased ACSL4 lactylation and expression were observed in rat MIRI myocardium, contributing to cardiomyocyte death and expanded infarcts. Wang et al. [96] further confirmed that H/R induces GPX4 lactylation at K218/K228, reducing its protein stability, increasing Fe^2+^, MDA and LDH levels, and promoting cardiomyocyte ferroptosis and infarct enlargement.

In summary, in MIRI, lactylation at sites such as H3K56 and Serpina3k exerts protective effects, whereas lactylation of Snail1, NLRP3, ACSL4, MDH2 and others produces deleterious outcomes. H3K18la exhibits cell-type-dependent dual regulation: in macrophages, it binds promoters of reparative genes and is beneficial, while in cardiomyocytes, it can bind promoters of pro-apoptotic or pro-hypertrophic genes to induce cell death. However, current research is largely limited by the fact that most lactylation events are observed only under the overall context of MIRI, and the distinct contributions of lactylation during the ischemic versus reperfusion phases remain insufficiently defined. Existing evidence suggests that lactylation associated with glycolytic upregulation during ischemia may, to some extent, maintain cell survival by promoting ATP generation. In contrast, persistent lactylation induced by high lactate levels during reperfusion may exacerbate injury by promoting inflammation and inducing acidosis. Furthermore, although ischemia and hypoxia trigger lactylation due to lactate elevation, lactylation is not merely an accompanying phenomenon in this process but rather acts as a functional executor, playing an indispensable role in MIRI. Studies have shown that when specific lactylation sites (e.g., MDH2 K241) are mutated and cannot undergo modification, myocardial injury is substantially reduced even under ischemic or high-lactate conditions. This indicates that the injury is mediated by lactylation itself rather than simply the direct consequence of ischemia. Additionally, by targeting the lactylation “writer” P300, lactylation levels—and thus downstream gene expression and I/R outcomes—can be modified without altering lactate concentrations. Therefore, future research should prioritize delineating the dynamic regulatory features of lactylation throughout MIRI, identifying its critical intervention time windows, and exploring strategies to steer its outcomes toward a beneficial end.

#### 4.1.3. Heart Failure (HF)

Heart failure is a complex clinical syndrome that represents the end stage of various cardiovascular diseases. Recent studies indicate that lactate-mediated lactylation plays important roles in HF pathogenesis and prognosis.

The cardiac sarcomere is the basic functional and structural unit of contraction in cardiomyocytes; its structural integrity is essential for systolic and diastolic function. α-myosin heavy chain (α-MHC) lactylation at K1897 is critical for maintaining sarcomeric structural stability and is dynamically regulated by intracellular lactate levels. Lactate accumulation promotes α-MHC K1897la, enhancing its interaction with titin and thereby preserving sarcomere integrity and contractile function; conversely, reduced lactate levels in HF decrease α-MHC lactylation, impair cardiac function and accelerate HF progression—thus, α-MHC lactylation appears cardioprotective [97]. Cardiomyocyte hypertrophy initially serves as a compensatory response to pressure overload but sustained hypertrophy leads to maladaptive remodeling, cell death and dilation, culminating in severe HF. Chen et al. [98] showed that METTL7B overexpression in cardiomyocytes and mouse hearts promotes m6A-mediated degradation of USP38 mRNA, reducing HDAC3 expression and thereby diminishing its de-lactylase activity; this increases H3K18la, downregulates β-MHC, ANP and BNP expression, attenuates remodeling, and improves cardiac function, delaying HF. Guo et al. [99] reported that in a transverse aortic constriction (TAC)-induced hypertrophy model, ACAA2 expression and its lactylation are significantly decreased, impairing fatty acid β-oxidation and mitochondrial oxidative phosphorylation, leading to metabolic dysfunction, pathological hypertrophy and worsened HF; while sodium lactate supplementation can restore ACAA2 lactylation, correct metabolic disturbance and alleviate hypertrophy, suggesting ACAA2 lactylation as a potential metabolic therapeutic target in HF.

The impact of lactylation on heart failure is not unidirectional but exhibits a distinct duality. It regulates opposing pathophysiological processes depending on the specific cell type and pathological context. As previously discussed, lactylation predominantly exerts protective effects within cardiomyocytes. In contrast, it mediates predominantly adverse effects in aortic valvular interstitial cells. This cell-type-specific disparity is particularly pronounced under conditions of myocardial energy deficiency and in the pro-calcific microenvironment of the valves.

Aortic valve calcification can lead to aortic stenosis, which increases cardiac workload, thereby inducing left ventricular remodeling and ultimately culminating in heart failure. Lactylation-driven valve calcification constitutes one of the pathological foundations of this process. Wu et al. [100] reported in the context of calcific aortic valve disease (CAVD) that lactate induces lactylation at the K42 site of aldolase A (ALDOA), which promotes the osteogenic differentiation of human aortic valvular interstitial cells (hVICs) and thereby drives the process of valvular calcification. Consistently, in an ApoE^−/−^ mouse model of CAVD induced by a high-fat diet, ALDOA K42la was also observed to promote aortic valve calcification, consequently increasing the risk of heart failure. Zhang et al. [101] demonstrated that H3K9 lactylation enhances expression of osteogenic genes BMP2 and RUNX2, aggravating valve calcification and HF progression. GAPDH lactylation activates a GAPDH-glycolysis-osteogenesis axis in hVICs, ApoE^−/−^ mice models, and clinical samples, driving valve calcification; notably, butyrate competitively inhibits GAPDH lactylation and blocks this pathological axis, proposing a new therapeutic avenue [102].

On the other hand, Zhao et al. [103] showed from TAC-induced cardiomyocytes and mouse models that enhanced glycolysis leads to lactate accumulation and increased histone lactylation, particularly H3K18la, which upregulates β-MHC, ANP and BNP to induce hypertrophy and fibrosis, thereby worsening cardiac function and HF. Although this finding appears to contradict the previously described beneficial role of H3K18la in heart failure, the discrepancy may originate from distinct upstream regulatory mechanisms governing lactylation. Specifically, the differential outcomes are likely influenced by the specific signaling context—namely, the METTL7B-USP38-HDAC3 regulatory axis versus glycolysis-driven lactylation—highlighting the functional diversity of the same epigenetic mark when embedded within different cellular signaling networks.

#### 4.1.4. Other Cardiovascular Conditions

Cardiomyocyte regeneration refers to restoration of injured myocardium via cardiomyocyte proliferation or progenitor cell differentiation, which fundamentally improves cardiac function and prognosis. Studies indicate that lactate-induced histone lactylation, particularly H3K18la, downregulates cell-cycle inhibitors p21 and p53 while upregulating cell-cycle promoters Ccnd1 and Cdk2, thereby releasing cardiomyocytes from cell-cycle arrest and promoting proliferation and regeneration [104]. Zhang et al. [105] further confirmed that neonatal mouse hearts retain robust regenerative capacity within 7 days after birth, correlating with higher lactylation levels; as lactylation declines, regenerative ability is lost. How does lactylation affect the regeneration of cardiomyocytes in mice? Mechanistically, lactylation enhances glycolytic enzyme activity and promotes expression of cell-cycle regulators NPM1 and SMC3 to sustain cardiomyocyte proliferation [105]. Thus, lactylation may serve as a key epigenetic mechanism regulating cardiac regeneration. Its potential as a novel target to induce cardiac regeneration holds promise for providing new therapeutic strategies for myocardial infarction and heart failure.

Aortic dissection (AD) is a highly lethal and rapidly progressive cardiovascular emergency whose pathogenesis is closely related to lactylation. Yu et al. [106] found markedly elevated protein lactylation in AD mouse models and clinical patient tissues, particularly in medial VSMCs. Lactylation of ATP5F1A at K531 reduces ATP synthase activity, causing mitochondrial structural and functional deficits, decreased ATP production and ROS accumulation, which promote a phenotypic switch of VSMCs from a contractile to a synthetic state and compromise vascular wall integrity, exacerbating AD progression [106]. Li et al. [107] also observed lactate accumulation-induced H3K18la enrichment at the p53 promoter in AD, promoting p53 expression and VSMC apoptosis, further contributing to AD. Targeting pathogenic lactylation events to slow AD progression is also an active area of investigation.

**Table 1:** Beneficial effects of lactylation modifications in vascular diseases.

Disease	Lactylation Site	Cell Type	Mechanism	Reference
Atherosclerosis	H3 (K18)	Macrophages	Promotes expression of repair genes, drives macrophage polarization toward M2 phenotype, reduces plaque area	[82]
	MeCP2 (K271)	ECs	Inhibits Ereg and blocks EREG/MAPK pathway, downregulating inflammatory cytokine expression	[16]
	MeCP2 (K271)	Macrophages	Promotes macrophage polarization to M2 type, enhances plaque stability	[83]
MIRI	H3 (K18)	Macrophages	Enhances anti-inflammatory and pro-angiogenic activity of monocyte–macrophage axis, promotes cardiac repair	[11]
	H3 (K56)	Cardiomyocytes	Alleviates myocardial ischemia–reperfusion injury	[88]
	Serpina3k (K351)	Cardiac fibroblasts	Inhibits WNT pathway, activates RISK/SAFE pathway, suppresses cardiomyocyte apoptosis, reduces infarct size	[15]
HF	α-MHC (K1897)	Cardiomyocytes	Enhances interaction with Titin, maintains sarcomere structure and contractile function	[97]
	H3 (K18)	Cardiomyocytes	Decreases β-MHC, ANP, BNP expression, alleviates cardiac remodeling	[98]
	ACAA2	Cardiomyocytes	Improves fatty acid β-oxidation and mitochondrial function, reduces cardiac hypertrophy	[99]
Cardiomyocyte regeneration	H3 (K18)	Cardiomyocytes	Downregulates cell-cycle-inhibitory genes, releases cell-cycle arrest, promotes cardiomyocyte proliferation and regeneration	[104,105]
Ischemic stroke	H3 (K18)	Microglia	Upregulates PLXNB2, drives anti-inflammatory phenotype conversion, inhibits neuronal apoptosis	[108]
	MeCP2 (K210, K249)	Neurons	Suppresses transcription of apoptotic genes, reduces neuronal apoptosis	[109]
SAH	H4 (K8)	Astrocytes	Inhibits A1 astrocyte polarization, decreases release of inflammatory cytokines	[110]

MIRI: Myocardial ischemia–reperfusion injury, HF: Heart failure, SAH: Subarachnoid hemorrhage, ECs: Endothelial cells, hVICs: human aortic valvular interstitial cells.

### 4.2. Cerebrovascular Diseases

#### 4.2.1. Ischemic Stroke

Ischemic stroke, caused by abrupt interruption of cerebral blood flow leading to ischemic and hypoxic neuronal death, accounts for the vast majority of stroke cases. Its fundamental etiology lies in cerebrovascular occlusion caused by thrombosis or embolism, which triggers a pathological process centered on the ischemic cascade and may even lead to cerebral ischemia–reperfusion injury.

In recent years, accumulating evidence has shown that lactate can act as a protective mediator in the central nervous system by exerting anti-inflammatory effects, limiting cerebral edema, promoting post-ischemic cerebrovascular regeneration, reducing infarct size, and alleviating ischemic brain injury [111,112]. Given lactate’s well-established protective role, a new question naturally arises: beyond acting directly as a signaling molecule, does lactate-mediated lactylation exert protective effects in cerebral ischemia? Subsequent studies have confirmed that lactylation indeed confers neuroprotection in this context. For example, Li et al. [108] found that lactate enhances H3K18la to upregulate PLXNB2 expression in microglia, promoting an anti-inflammatory phenotype and release of IL-10 and TGF-β, suppressing neuronal apoptosis and aiding functional recovery. Sun et al. [109] revealed that under cerebral ischemia, lactylation of MeCP2 at K210 and K249 causes MeCP2 to accumulate at promoters of apoptosis-related genes Pdcd4 and Pla2g6, repressing their transcription and reducing neuronal apoptosis, thereby conferring neuroprotection.

However, more evidence points to deleterious effects. During cerebral ischemia, anaerobic glycolysis is enhanced and lactate accumulates, promoting lactylation of phospholipase B domain containing 1 (PLBD1) at K155, stabilizing the protein and activating the NLRP3 inflammasome to exacerbate pyroptosis and inflammation [113]. Zhang et al. [114] reported that in rat stroke models and OGD/R cell models, lactylation of lymphocyte cytosolic protein 1 (LCP1) increases its stability and promotes immune cell infiltration into the brain, amplifying inflammation and tissue damage. Moreover, ischemia-induced upregulation of miR-125a-5p targets SMEK1 to reduce PDH activity, leading to lactate accumulation and enhanced H3K9la, which directly promotes transcription of glycolytic genes LDHA and HIF-1α, forming a “lactate–H3K9la–glycolysis” positive feedback loop that drives microglia toward a pro-inflammatory M1 phenotype and expands neuroinflammation and tissue injury [115]. Under ischemia/hypoxia, protein lactylation in microglia is broadly elevated; lactylation of cGAS at K162 increases its stability and activates the cGAS-STING pathway to promote M1 polarization and aggravate neuroinflammation [116].

Astrocytes, as central regulators in the CNS, are also significantly affected by lactylation during cerebral ischemia. Xiong et al. [117] reported that augmented glycolysis in astrocytes during ischemic stroke increases lactate production, and substantial lactate-entering neurons promotes protein lactylation, activating NF-κB, p53 and Notch signaling to exacerbate inflammation and neuronal death, as well as upregulating A1 astrocyte markers GFAP and Serping1, thereby increasing neurotoxicity. Meanwhile, H3K18la during brain ischemia can also induce NSUN2 expression and promote astrocyte A1 polarization [118]. Zhou et al. [119] further showed that lactylation of ARF1 at K73 in astrocytes upregulates ARF1, disrupts vesicle trafficking, inhibits transfer of functional mitochondria to neurons, impairs neuronal energy metabolism and worsens brain injury.

Ischemic stroke is a dynamic process with acute, recovery and chronic phases. Lactylation exerts divergent effects depending on the microenvironment at different times (e.g., lactate concentration, pH): in the acute phase, severe hypoxia and acidosis lead to massive lactate accumulation and high-concentration lactylation that tends to drive pro-inflammatory and pro-apoptotic responses (e.g., activation of NLRP3, cGAS-STING), worsening injury; in the recovery phase, the microenvironment stabilizes and lactate levels decrease, potentially initiating protective lactylation that limits inflammation and promotes repair. While this general pattern holds for many settings, exceptions exist—for example, acute ischemia may also see lactylation suppress neuronal apoptosis in some contexts. The differences likely reflect ischemic severity and cell-type specificity: astrocytic lactylation often appears deleterious, microglial lactylation can be double-faced, and neuronal lactylation may lean toward protection. However, precise regulatory networks and intercellular communication mediated by lactylation remain to be elucidated. Identifying the key nodes that switch lactylation from protective to harmful may provide novel therapeutic strategies for ischemic stroke.

#### 4.2.2. Hemorrhagic Stroke

Hemorrhagic stroke is mainly divided into intracerebral hemorrhage (ICH) and subarachnoid hemorrhage (SAH) according to the site of bleeding; these conditions have an abrupt onset, rapidly progressive course and generally poor prognosis.

Studies show that SAH markedly enhances astrocytic glycolysis, leading to abundant lactate generation that meets neuronal and other brain cell energy demands and, by inhibiting early A1 astrocyte polarization after SAH, may exert neuroprotective effects [120]. Zhang et al. [110] found elevated H4K8la in astrocytes after SAH in mice; bromodomain-containing protein 4 (BRD4) recognizes H4K8la and suppresses A1 astrocyte polarization, reducing release of IL-1β, TNF-α and IL-6, inhibiting neuronal apoptosis and promoting functional recovery.

In addition to the early protective mechanisms in SAH, lactylation can also mediate neuronal damage. Lactylome analysis of SAH mouse brains revealed broad lactylation across neurons, astrocytes and microglia; lactylation was prominent at β-arrestin1 (ARRB1) K195, which promotes ARRB1 interaction with S100A9 and inhibits PGC-1α/NRF1 signaling, causing mitochondrial dysfunction and neuronal apoptosis [121].

In contrast to SAH, lactylation in the acute phase of ICH predominantly contributes to neuronal injury. Sun et al. reported massive lactate accumulation within 24 h after ICH in mice, accompanied by significant elevation of neuronal H3K14la. Combined ChIP-seq and RNA-seq analyses showed H3K14la enrichment at the ATP2B2 (PMCA2) gene region and suppression of its expression; PMCA2 encodes a calcium pump responsible for extruding cytosolic Ca^2+^, and its inhibition activates calpain, causing intracellular Ca^2+^ overload and inducing neuronal ferroptosis, worsening injury [122]. Additionally, ICH induces lactylation of METTL3 in neurons, enhancing METTL3 expression and its mediated m6A methylation, stabilizing transferrin receptor (TFRC) transcripts and increasing iron uptake, further promoting ferroptosis [123]. Zhang et al. [124] also confirmed that LDHA promotes histone lactylation at the p53 promoter, enhancing p53 transcriptional activity, upregulating pro-apoptotic Bax and cleaved caspase-3 and downregulating anti-apoptotic Bcl-2, ultimately exacerbating neuron apoptosis and brain injury after hemorrhage.

Overall, current studies on lactylation in hemorrhagic stroke are mainly limited to the acute phase (within 24 h) and exhibit subtype specificity and duality: in early SAH, H4K8la may rapidly address energy crisis and inhibit acute inflammation as a compensatory neuroprotective mechanism, while ARRB1 lactylation mediates mitochondrial dysfunction and neuronal damage. This detrimental effect coexists with the protective role of H4K8la, collectively shaping the prognosis of SAH. In acute ICH, lactylation predominantly promotes pathological processes such as Ca^2+^ overload and ferroptosis, manifesting as adverse effects. Differences between subtypes likely arise from distinct initial injuries: global ischemia in SAH may preferentially trigger compensatory protective mechanisms, whereas focal hematoma, iron overload and severe inflammation in ICH create a strongly pro-injury microenvironment that amplifies harmful lactylation effects. Therefore, future research should not only address critical gaps during the subacute and recovery stages, but they should also prioritize comparing the lactylation-regulated networks across different stroke subtypes, which is crucial for developing targeted therapeutic strategies.

### 4.3. Other Vascular Diseases

Although abundant evidence supports a dual role of lactylation in cardiovascular and cerebrovascular diseases—promoting tissue repair by reducing inflammation and apoptosis yet exacerbating disease progression at specific stages—protective lactylation has not been demonstrated in certain vascular conditions such as diabetes-associated vascular disease and preeclampsia, where current data more often indicate pathogenic roles.

#### 4.3.1. Diabetes-Associated Vascular Disease

Metabolic dysregulation is central to diabetic vascular complications. In diabetes, especially under hyperglycemic conditions, enhanced glycolysis increases lactate levels, driving lactylation that plays critical roles in disease progression. Zhu et al. [125] found that in diabetic vascular calcification mouse and VSMC models, high-glucose-induced calcifying environments cause lactate accumulation, and ChIP-qPCR reveals p300-mediated H3K18la enrichment at the CHI3L1 promoter, upregulating CHI3L1. Transcriptome analysis indicated CHI3L1 activates the IL-13–IL-13Ra2–JAK1–STAT3 axis, increasing osteogenic markers BMP2 and RUNX2 and promoting osteogenic differentiation of VSMCs, accelerating diabetic arterial calcification. In diabetic retinopathy (DR), elevated H3K18la was also observed; H3K18la upregulates RNA demethylase FTO, disrupting m6A homeostasis and stabilizing CDK2 mRNA, driving aberrant retinal vascular endothelial proliferation, migration and neovascularization, worsening DR [126]. As a common microvascular complication of diabetes, the core pathological processes of Diabetic Kidney Disease (DKD) have been shown by multiple studies to be influenced by lactylation. Streptozotocin-induced mouse models and high-glucose-treated podocytes show massive lactate accumulation leading to LARS1 K970 lactylation, which activates mTORC1 signaling, inhibits autophagy and promotes podocyte apoptosis. Importantly, reducing lactylation levels alleviates glomerular structural damage and slows DKD progression [127].

#### 4.3.2. Preeclampsia

Preeclampsia is a pregnancy-specific progressive systemic vascular disorder that threatens maternal–fetal health; its pathogenesis is closely related to placental hypoxia and metabolic reprogramming [128], with lactylation playing key regulatory roles. Lu et al. [129] reported significantly increased histone and non-histone lactylation in placentas from preeclampsia patients. Mechanistically, upregulation of HK2 in placental vascular endothelial cells enhances glycolysis and induces H3K18la, activating NLRP3 inflammasome and caspase-1-mediated pyroptosis, leading to placental endothelial injury. Furthermore, RNA-seq and ChIP-qPCR demonstrated H3K18la enrichment at promoters of fibrosis-related genes Fibronectin 1 and SERPINE1, upregulating their transcription; Fibronectin 1 overexpression promotes extracellular matrix deposition and placental fibrosis, while SERPINE1 inhibits fibrinolysis and increases thrombosis risk, exacerbating vascular remodeling defects [130]. Li’s team [131] also confirmed that H3K18la promotes transcription of pro-senescence gene GADD45A, triggering p16-dependent trophoblast premature senescence and subsequent placental dysfunction. Xu et al. [132] found increased PKM2 K305 lactylation in preeclamptic placentas, which negatively regulates PKM2 enzymatic activity, impairs glycolysis and energy metabolism, and inhibits trophoblast proliferation. Beyond these mechanisms, lactylation has been implicated in the progression of preeclampsia through the regulation of mitochondrial function. For example, the lactylation of mitochondrial chaperone Hsp60 at K469 and K473 promotes mitochondrial fission and oxidative stress, driving trophoblast apoptosis and placental vascular remodeling defects [133].

#### 4.3.3. Others

Arterial calcification is characterized by abnormal calcium salt deposition in the arterial media, causing stiffening and loss of elasticity [134]. Nuclear receptor subfamily 4 group A member 3 (NR4A3) is emerging as a critical regulator not only in the progression of atherosclerosis but also in the pathogenesis of arterial calcification. Ma et al. [135] found that NR4A3 binds promoters of glycolytic enzyme genes ALDOA and PFKL to enhance their transcription, increasing glycolytic flux and lactate production, as well as inducing H3K18la; ChIP-qPCR confirmed H3K18la enrichment at the Phospho1 promoter, promoting its expression and accelerating inorganic phosphate release and calcium–phosphate deposition, thereby promoting vascular calcification. In oxygen-induced retinopathy models, lactate-driven histone lactylation, notably H3K9la and H3K18la, activates PRMT5 transcription and upregulates glycolytic enzymes LDHA and PFKFB3, forming a positive feedback loop that sustains pathological retinal neovascularization; inhibiting histone lactylation effectively attenuates pathological angiogenesis and offers a potential intervention for ischemic retinopathies [136] (Table 2).

## 5. Translational Prospects: From Bench to Bedside

A deeper understanding of the lactate–lactylation process in vascular diseases holds great promise for clinical translation, mainly in developing novel biomarkers and innovative therapeutic strategies.

### 5.1. Lactylation as a Novel Biomarker

Lactylation and related molecules show emerging potential as clinical biomarkers across multiple vascular diseases for early diagnosis, severity assessment and prognosis prediction.

In AD, multi-omics analyses identified 12 core lactylation-related genes (e.g., LDHA, SPP1, SLC16A3) with excellent discriminatory power (AUC ≥ 0.8893) [137]. Similarly, in carotid atherosclerosis, transcriptomic and single-cell sequencing uncovered three differentially expressed lactylation-associated genes—SOD1, DDX42 and PDLIM1—that differ significantly between plaque and normal tissue, highlighting potential as a diagnostic biorender [138]. Bioinformatic analyses in heart failure also revealed multiple differentially expressed lactylation-related genes with potential clinical utility. Molecular subtyping based on lactylation-related gene expression can stratify AMI patients into high- and low-risk subgroups for precision therapy; genes such as SLC7A7, PYGL, SAT1 and AMPD2 may modulate monocyte activation and immune responses and serve as early diagnostic and prognostic markers in AMI [139].

### 5.2. Therapeutic Strategies Targeting the Lactate–Lactylation Process

Given the double-edged roles of the lactate–lactylation process in regulating vascular cell function and its involvement in atherosclerosis, HF, stroke and other vascular diseases, targeting this process is an attractive therapeutic direction. Current preclinical strategies fall into four major categories:

Inhibiting lactate production (targeting LDH): LDHA is the critical rate-limiting enzyme that catalyzes lactate production. Small-molecule LDHA inhibitors such as oxamate and FX11 show therapeutic promise across vascular disease models: they can reverse Aortic dissection (AD) pathology, reduce lesion severity and increase survival in AD mouse models [106], as well as promote extracellular mitochondrial and ATP release to mediate neuroprotection [119,121].

Blocking lactate transport (targeting MCTs): Monocarboxylate transporters MCT1/MCT4 mediate lactate transmembrane transport; inhibitors like AZD3965 can disrupt lactate shuttling and modulate lactylation. Targeting MCT1/MCT4 effectively mitigates diabetic arterial calcification [125], and targeting MCT4 in atherosclerosis displays anti-inflammatory and reparative effects, supporting their vascular protective potential [82].

Modulating lactylation “writers” (targeting p300/CBP): p300/CBP, a major acetyltransferase, can also use lactyl-CoA to transfer lactyl groups onto lysine residues. p300/CBP inhibitors such as A-485 and C646 competitively bind the catalytic site to inhibit lactyl group transfer, decreasing pro-inflammatory gene expression and pathological angiogenesis [126]. Notably, inhibition of p300 may reduce lactylation to an extent that paradoxically aggravates inflammation and accelerates atherosclerosis [82], highlighting the need for nuanced interventions.

Modulating lactylation “erasers” (targeting HDACs): Histone deacetylases (HDAC1-3) exhibit de-lactylase activity. HDAC inhibitors can raise intracellular lactylation levels and have been shown to exacerbate neuronal ferroptosis [122]; their potential to selectively activate protective lactylation pathways remains unclear and warrants further study.

### 5.3. Unresolved Questions and Future Directions

Despite the explosive growth of lactylation research in just a few years, translating its double-edged sword effect in vascular diseases into precise therapeutic strategies still faces numerous challenges. The following key scientific questions urgently await answers:

Identifying specific regulatory enzymes: Current “writers” (p300/CBP) and “erasers” (HDACs) are mostly broad in substrate specificity. Are there more specific lactyltransferases or delactylases suited as drug targets? Discovering such enzymes is a prerequisite for precise intervention.

Mapping dynamic lactylomes: Comprehensive, time-resolved lactylation proteomes across vascular diseases and disease stages are lacking. Advanced mass spectrometry to build these dynamic atlases is essential to identify disease-specific targets and biomarkers.

Deciphering the “modification code”: Lactylation does not act in isolation but cross-talks with acetylation, ubiquitination and other post-translational modifications. Are these interactions competitive, cooperative or antagonistic? Understanding this code and how combined modifications dictate cell fate is crucial to comprehending its precise regulatory mechanisms.

Establishing functional “concentration thresholds”: The beneficial or harmful outcomes of lactylation depend on lactate concentration. What precise lactate thresholds trigger different biological effects? How are these thresholds regulated in vivo within complex microenvironments? Answering these questions is vital for distinguishing demarcation between physiological and pathological states.

Achieving precise targeted interventions: Given lactylation’s dual role, how can pathological lactylation be specifically suppressed while preserving or enhancing physiological protective lactylation? This demands a paradigm shift from broad-spectrum inhibitors to next-generation agents with precision for specific proteins, cell types, or organelles.

## 6. Conclusions

From a once-dismissed metabolic byproduct to a central hub linking cellular metabolism and gene regulation, lactate and lactate-mediated lactylation have undergone a paradigm shift in life-science research and have reshaped our understanding of vascular disease mechanisms. This review systematically elucidates that the lactate–lactylation process, as a key metabolic sensor and effector, participates in processes such as atherosclerosis, heart failure, stroke and diabetes-related vascular disease through complex, context-dependent bidirectional regulation. However, the precise differentiation and regulation of its protective versus pathological roles require an in-depth understanding of the lactate–lactylation process. Unquestionably, investigating this process represents one of the most exciting frontiers in vascular disease research. Whether through developing high-sensitivity lactylation-based biomarkers for early diagnosis and precision stratification or designing novel drugs targeting LDH, MCTs or lactylation enzymes themselves, translating lactylation research from bench to bedside will be a major mission in translational medicine in the coming years and is likely to improve diagnostic precision and therapeutic efficacy for vascular diseases.

## Figures and Tables

**Table 2:** Adverse effects of lactylation modifications in vascular diseases.

Disease	Lactylation Site	Cell Type	Mechanism	Reference
Atherosclerosis	H3 (K18)	ECs	Promotes SNAI1 expression, induces endothelial-to-mesenchymal transition	[84]
	H3 (K18)	Macrophages	Promotes METTL3 expression, accelerates SLC7A11 mRNA degradation via YTHDF2-dependent m6A, induces ferroptosis	[10]
	H4 (K12)	VSMCs	Enriches at SASP gene promoters, promotes SASP expression, exacerbates VSMC senescence	[85]
MIRI	Snail1	ECs	Upregulates TGF-β expression and activates TGF-β/Smad2 pathway, promotes EndMT and myocardial fibrosis	[93]
	NLRP3 (K245)	Cardiomyocytes	Enhances NLRP3 stability and activation, promotes IL-1β and IL-18 release, exacerbates pyroptosis	[89]
	H3 (K18)	Cardiomyocytes	Enriched at TRPM7 promoter, promotes TRPM7 expression, causes Ca^2+^ overload and apoptosis	[90]
	H3 (K18)	Cardiomyocytes	Promotes YTHDF2 transcription and upregulates G3BP1, leading to hypertrophy and apoptosis	[91]
	MDH2 (K241) ACSL4 (K83) GPX4 (K218, K228)	Cardiomyocytes	Promotes myocardial cell ferroptosis and aggravates MIRI	[94,95,96]
HF	H3 (K9) ALDOA (K42) GAPDH	hVICs	Stimulates the differentiation of hVICs into osteoblast-like cells and promotes valve calcification	[100,101,102]
	H3 (K18)	Cardiomyocytes	Upregulates β-MHC, ANP, BNP, induces hypertrophy and fibrosis, worsens cardiac function	[103]
Aortic dissection	ATP5F1A (K531)	VSMCs	Reduces ATP synthase activity, impairs mitochondrial function, promotes VSMC synthetic switch and aortic dissection progression	[106]
	H3 (K18)	VSMCs	Enriches at p53 promoter, promotes p53 expression and VSMC apoptosis, facilitating aortic dissection	[107]
Ischemic stroke	PLBD1 (K155)	Microglia	Stabilizes PLBD1, activates NLRP3 inflammasome, promotes pyroptosis and inflammation	[113]
	LCP1	Neurons	Increases LCP1 stability, promotes immune cell infiltration and inflammation, worsening tissue damage	[114]
	H3 (K9)	Microglia	Enhances glycolytic gene transcription, drives M1 polarization and neuroinflammation	[115]
	cGAS (K162)	Microglia	Activates cGAS-STING pathway, promotes M1 polarization	[116]
	H3 (K18)	Astrocytes	Promotes NSUN2 expression, activates A1 astrocyte polarization	[118]
	ARF1 (K73)	Astrocytes	Upregulates ARF1, disrupts vesicular transport, inhibits mitochondrial transfer to neurons, thus worsening energy deficit	[119]
ICH	H3 (K14) METTL3	Neurons	Promote neuronal ferroptosis and exacerbate neuronal damage	[122,123]
	H3 (K18)	Neurons	Enhances p53 activity, upregulates pro-apoptotic proteins, promoting neuronal apoptosis	[124]
SAH	ARRB1 (K195)	Neurons, Astrocytes, Microglia	Promotes ARRB1 interaction with S100A9, inhibits PGC-1α/NRF1 signaling, causes mitochondrial dysfunction and neuronal apoptosis	[121]
Diabetic arterial calcification	H3 (K18)	VSMCs	Upregulates CHI3L1 expression, activates IL-13/IL-13Ra2/JAK1/STAT3 axis, promotes osteogenic differentiation and calcification	[125]
Diabetic retinopathy	H3 (K18)	Retinal endothelial cells	Upregulates FTO expression, disrupts m6A homeostasis, stabilizes CDK2 mRNA, promotes endothelial proliferation and neovascularization	[126]
Diabetic kidney disease	LARS1 (K970)	Podocytes	Activates mTORC1, inhibits autophagy and promotes podocyte apoptosis	[127]
Preeclampsia	H3 (K18)	Placental endothelial cells	Activates NLRP3 inflammasome and caspase-1 pyroptosis, causing placental endothelial injury	[129]
	H3 (K18)	Trophoblasts	Enriches at Fibronectin 1 and SERPINE1 promoters, upregulates fibrosis genes and impairs vascular remodeling	[130]
	H3 (K18)	Trophoblasts	Promotes GADD45A transcription, induces trophoblast premature senescence	[131]
	PKM2 (K305)	Trophoblasts	Inhibits PKM2 activity, impairs glycolysis and trophoblast proliferation	[132]
	Hsp60 (K469, K473)	Trophoblasts	Promotes mitochondrial fission, oxidative stress and trophoblast apoptosis, hindering placental vascular remodeling	[133]
Arterial medial calcification	H3 (K18)	Vascular-associated cells	Upregulates Phospho1, accelerates inorganic phosphate release and calcium–phosphate deposition	[135]
Pathological retinal neovascularization	H3 (K9), H3 (K18)	Endothelial cells	Activates PRMT5 transcription, forming a positive feedback loop sustaining pathological angiogenesis	[136]

MIRI: Myocardial ischemia–reperfusion injury, HF: Heart failure, ICH: Intracranial hemorrhage, SAH: Subarachnoid hemorrhage, ECs: Endothelial cells, VSMCs: Vascular smooth muscle cells.

## Data Availability

The article is based on previously published studies, which are cited in the reference list.

## Abbreviations

The following abbreviations are used in this manuscript:

2-DG	2-deoxy-D-glucose
α-MHC	α-myosin heavy chain
ACSS2	acetyl-CoA synthetase 2
AD	Alzheimer’s disease/Aortic dissection
ALDH2	aldehyde dehydrogenase 2
ALDOA	aldolase A
ARRB1	β-arrestin1
BRD4	bromodomain-containing protein 4
CAVD	calcific aortic valve disease
DHAP	dihydroxyacetone phosphate
DKD	Diabetic Kidney Disease
DR	diabetic retinopathy
EAU	experimental autoimmune uveitis
ECs	endothelial cells
EndMT	endothelial-to-mesenchymal transition
FASN	fatty acid synthase
G6PD	glucose-6-phosphate dehydrogenase
GCGR	glucagon receptor
GLO1	glyoxalase 1
GLP1R	glucagon-like peptide-1 receptor
HF	Heart Failure
HK2	hexokinase 2
HSCs	hepatic stellate cells
ICH	intracerebral hemorrhage
IFN-1	interferon 1
IGF2BP2	insulin-like growth factor 2 mRNA-binding protein 2
Kla	lysine lactylation
LCP1	lymphocyte cytosolic protein 1
LDHA	lactate dehydrogenase A
LGSH	lactoyl-glutathione
MCT	monocarboxylate transporter
MDH2	malate dehydrogenase 2
MGO	methylglyoxal
MI	Myocardial infarction
MIRI	myocardial ischemia–reperfusion injury
MPC1	mitochondrial pyruvate carrier 1
MeCP2	methyl CpG binding protein 2
NAFLD	non-alcoholic fatty liver disease
NR4A3	Nuclear receptor subfamily 4 group A member 3
PBMCs	peripheral blood mononuclear cells
PD-L1	programmed death-ligand 1
PFK1	phosphofructokinase-1
PHB2	prohibitin 2
PKM2	pyruvate kinase M2
PLBD1	phospholipase B domain containing 1
PPP	the pentose phosphate pathway
PTM	post-translational modification
RA	rheumatoid arthritis
SAH	subarachnoid hemorrhage
SASP	senescence-associated secretory phenotype
SCI	spinal cord injury
SF3B1	splicing factor 3B subunit 1
SLE	systemic lupus erythematosus
SLG	S-D-lactoylglutathione
TBI	traumatic brain injury
TFRC	transferrin receptor
TME	tumor microenvironment
VSMCs	vascular smooth muscle cells
ZGA	zygotic-genome activation
hVICs	human aortic valvular interstitial cells
